# Distribution of radiocarbon in sediments of the cooling pond of RBMK type Ignalina Nuclear Power Plant in Lithuania

**DOI:** 10.1371/journal.pone.0237605

**Published:** 2020-08-17

**Authors:** Rūta Barisevičiūtė, Evaldas Maceika, Žilvinas Ežerinskis, Justina Šapolaitė, Laurynas Butkus, Jonas Mažeika, Vytautas Rakauskas, Laurynas Juodis, Andrius Steponėnas, Rūta Druteikienė, Vidmantas Remeikis

**Affiliations:** 1 State Research Institute Center for Physical Sciences and Technology, Vilnius, Lithuania; 2 State Research Institute Nature Research Centre, Vilnius, Lithuania; University of Florida, UNITED STATES

## Abstract

The vertical distribution of radiocarbon (^14^C) was examined in the bottom sediment core, taken from Lake Drūkšiai, which has served as a cooling pond since 1983 for the 26 years of the Ignalina Nuclear Power Plant (INPP) operation using two RBMK-1500 reactors (Russian acronym for”Channelized Large Power Reactor”). ^14^C specific activity was measured in alkali-soluble and -insoluble fractions of the sediment layers. Complementary measurements of the ^210^Pb and ^137^Cs activity of the samples provided the possibility to evaluate the date of every layer formation, covering the 1947–2013 period. In addition, ^14^C distribution was examined in the scales of pelagic fish caught between 1980 and 2012. Our measurements reveal that, during the period 1947–1999, the radiocarbon specific activity in both fractions exhibits a parallel course with a difference of 5 ± 1 pMC (percent of modern carbon) being higher in alkali-soluble fraction, although ^14^C specific activity in both fractions increased by 11.4–13.6 pMC during the first 15 years of plant operation. However, during the 2000–2009 period, other than previously seen, a dissolved inorganic carbon (DIC) → aquatic primary producers → sediments ^14^C incorporation pattern occurred, as the radiocarbon specific activity difference between alkali-soluble and -insoluble fractions reached 94, 25, and 20 pMC in 2000, 2006, and 2008, respectively. Measurements in different sediment fractions allowed us to identify the unexpected organic nature of ^14^C contained in liquid effluences from the INPP in 2000–2009. The discrepancy between ^14^C specific activity in fish scales samples and DIC after 2000 also confirmed the possibility of organic ^14^C contamination. Possible reasons for this phenomenon might be industrial processes introduced at the INPP, such as the start of operation of the cementation facility for spent ion exchange resins, decontamination procedures, and various maintenance activities of reactor aging systems and equipment.

## Introduction

Recent studies show that radiocarbon (^14^C) can be used as a tracer for the evaluation of pollution from nuclear facilities and for tracing the carbon cycle and pathways in the environment [1–6]. Variations in the ^14^C/^12^C ratio in biota samples and bottom sediments can provide information about global processes and the influence of local human activities [7–13]. The radiocarbon, together with several isotopes of noble gasses such as ^41^Ar, ^85m^Kr, ^87^Kr, ^88^Kr, ^133^Xe, ^135^Xe, and ^135m^Xe, is one of the main radionuclides discharged to the environment by the nuclear industry and produced at a constant rate in the upper atmosphere. The amounts of ^14^C produced by different reactor types vary considerably. In particular, this depends on the materials of a neutron moderator, the composition of the reactor fuel and constructions, and the concentration of the target nuclei for the activation in these constructions. UNSCEAR [14] reported the average-normalized ^14^C release rates in the atmosphere for the period of 1990–1994 from various types of nuclear power reactors (in TBq/(GW_e_/y)): CANDU (1.6) > RBMK (1.3) > BWR (0.51) > PWR (0.22). The gaseous discharges from boiling water reactors (BWRs, RBMKs) and heavy water reactors (HWRs, CANDU type), as well as gas-cooled reactors (HTGR, AGR, Magnox), are mainly carbon dioxide ^14^CO_2_, whereas discharges from pressurized water reactors (PWRs) are dominated by hydrocarbons such as methane or ethane [15–25].

The Ignalina Nuclear Power Plant (INPP), situated in the northeast of Lithuania, operated two RBMK-1500 units (design electric power 1500 MW_e_): Unit 1 started operation in December 1983 and was shut down on December 31, 2004, whereas Unit 2 started operation in August 1987 and was shut down on December 31, 2009.

*Pinus sylvestris* (Linnaeus, 1753) ring measurements (samples were collected at a distance of a few kilometers from the INPP) showed an increase in ^14^C concentrations by 3–6 pMC during the first 14 years of operation (1983–1997) when no maintenance works of the reactors were needed [26–28]. During the operational period of 1998–2003, increased ^14^C specific activity values up to 6–14 pMC coincided with the replacement events of the zirconium alloy tubes of the fuel channels in both units of the INPP [27]. During the replacement procedure, the reactor graphite stack cavity is opened, and additional ^14^CO_2_ is expected to be released to the ventilation system of the plant.

The INPP uses Lake Drūkšiai as a cooling pond by the closed cooling loop and for technological water supply, as well as for controlled industrial drainage discharges from the plant. For a period of 1983–1999, ^14^C specific activity in dissolved inorganic carbon (DIC) from the surface water of the lake did not exceed the measured values in River Smalva entering the lake. However, ^14^C specific activity in DIC from Lake Drūkšiai increased in 2002–2006 with a ^14^C excess of 30–35 pMC (corresponding to the annual aqueous release of ~10^8^ Bq/y) [29].

The aim of this work is to evaluate the anthropogenic impact on the radiocarbon cycling in Lake Drūkšiai system using ^14^C specific activity measurements in the lake sediments. The impact of the INPP operation on the aquatic environment was assessed by comparing the evaluated ^14^C specific activity baseline levels assuming no INPP impact with the obtained new data of the radiocarbon measurement series in pelagic fish scales and the bottom sediment core layers for the whole INPP operation period. This new information on ^14^C excess in the aquatic environment is supplemented by previous measurements of tree rings obtained from *P*. *sylvestris* trees in the INPP vicinity and background areas [27], as well as radiocarbon analysis of DIC [29].

## Materials and methods

### Site description

**Fig 1:**
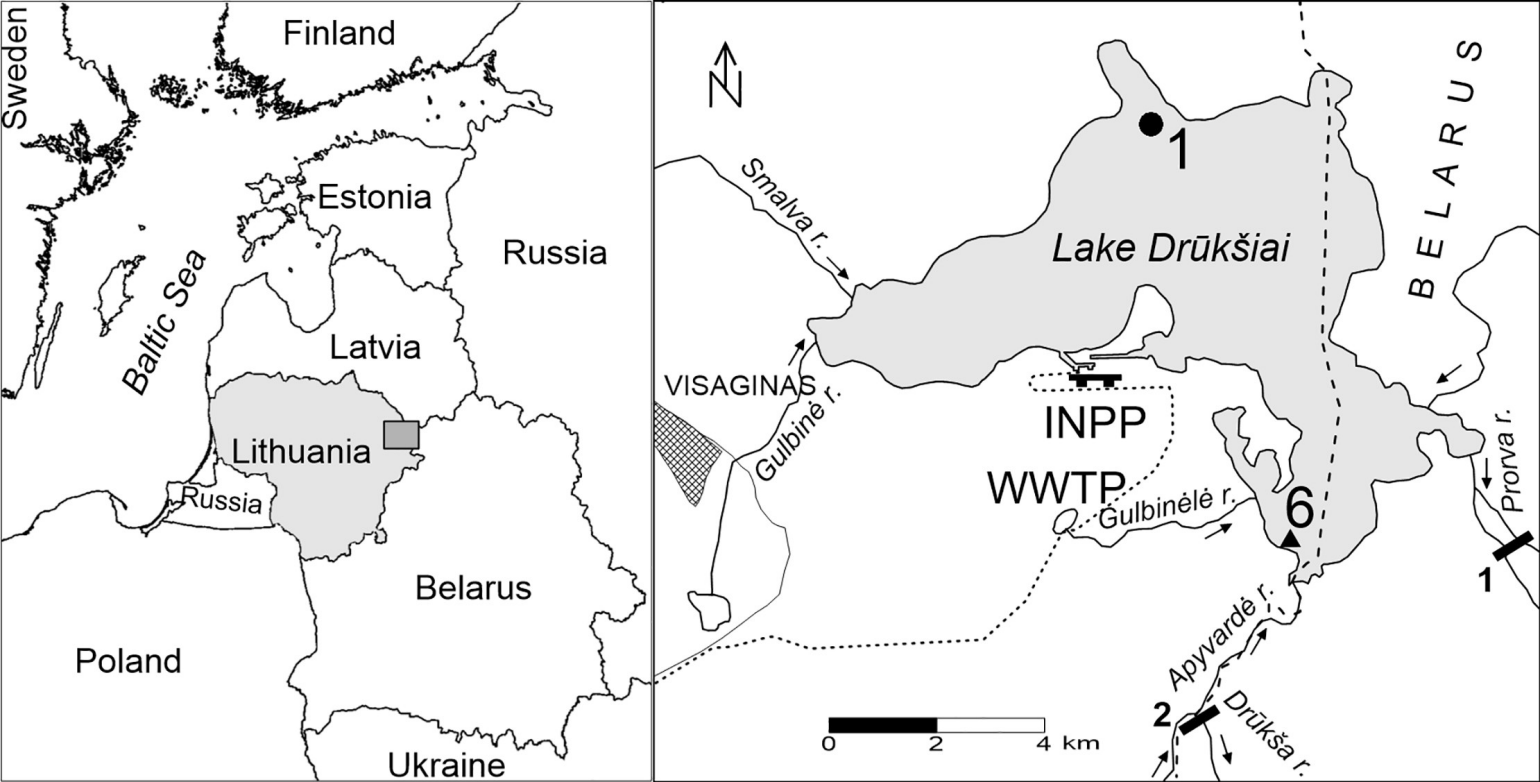
Location of Lake Drūkšiai and sampling sites. The sediment core site (station No 1) and water sampling site (station No 6) are marked by a circle and a triangle, respectively. The hydroelectric power plant (HEPP) and the dam on Drūkša river are marked by black bars 1 and 2, respectively. WWTP indicates location of the waste water treatment plant situated nearby a small lake connected to Lake Drūkšiai by Gulbinėlė rivulet. Industrial rain drainage system of Visaginas town via Smalva and Gulbinėlė streams is connected to Lake Drūkšiai. The short-dashed line means local railway route from regional line to INPP site.

Lake Drūkšiai is 14.3 km long, 5.3 km wide, and up to 33 m deep. The area of its water surface is 49 km^2^ and has a water volume of 0.37 km^3^. Lake Drūkšiai is situated in Northeastern Lithuania near the borders with Belarus and Latvia and is the biggest lake in Lithuania (Fig 1). Lake Drūkšiai is a flow-through lake with 11 small streams flowing in and one stream flowing out which has a water-level regulating dam. The lake is characterized by a relatively slow (3–4 y) water exchange rate and a high areal diversity of bottom sediments. For 26 years, Lake Drūkšiai served as a cooling basin for the INPP. The nutrient load from the town of Visaginas (founded for workers of the INPP) and the increase in water surface temperature, altering vertical thermal stratification, have caused changes in the trophic state of the lake from (oligo) mesotrophic to almost eutrophic [30]. In order to recover the key environmental changes that occurred in the lake and its environs, the sediment sampling site (Sampling Station No. 1, 55^o^38’49” N; 26^o^35’07” E) of this study was selected at 29 m depth and was close to the deepest depression of Lake Drūkšiai (Fig 1). The 62 cm length sediment core was collected in December 2013 from ice surface using a Kajak gravity corer with acrylic sample tube. The core was sectioned in 1 cm slices *in situ* using piston rod immediately after sampling. Samples in containers were transported to the laboratory and stored at -40°C until analysis. The characteristic for this lake depression are the highest recent sedimentation rate and finest sediments classified as muddy clay based on LOI and grain size analysis [31,32]. The clastic mineral material was dominating in these sediments with a concentration of carbonates and biogenic material ranging within 8.2–21.1% and within 19.6–32.6%, respectively [32].

### ^137^Cs, ^210^Pb, and ^214^Pb measurements in sediment samples

Sediment chronology for the last 66 years was determined using the ^210^Pb technique supported by anthropogenic ^137^Cs as a chronostratigraphic marker. Samples were assessed by gamma-ray spectrometry (measurement time 24–72 h). Activity of ^210^Pb (at 46.5 keV), ^226^Ra (from ^214^Pb and ^214^Bi at 295, 352, and 609 keV), and ^137^Cs (at 661 keV) isotopes were measured simultaneously using an HPGe well-type GWL-120-15-LBAWT detector (resolution 2.25 keV at 1.33 MeV) at the Nature Research Centre (Vilnius). Measurements were carried out in standard geometry, with 3 mL vials fitting to dimensions of the well-type detector. The mass of the samples was usually in the range of 2 to 4 g, but for a few top very fluffy samples it was approx. 1 g. The gamma spectrometric system was calibrated for counting efficiency using commercially available multi radionuclides standard sources and reference solutions for different densities and filing heights with the selected matrix as described in detail [31]. Uncertainties (±2 σ) of the radionuclide activity in the samples and the quality assurance procedures were evaluated by the GammaVision-32 software. The detection limit for the counting time of 200,000s was about 0.014 Bq for ^137^Cs, 0.065 Bq for ^210^Pb and 0.021 Bq for ^214^Pb, while the measurement errors did not exceed 8%, 15%, and 20% for ^137^Cs, ^210^Pb, and ^214^Pb, respectively.

The constant rate of the ^210^Pb supply (CRS) model [32–35] with some modifications [9] was used. The initial data on the activity concentration of ^210^Pb (total), ^214^Pb, and ^137^Cs in the sediment core versus depth with 1 cm resolution are given in S1 Fig.

The specific activity of the total ^210^Pb exponentially decreased from 360–460 Bq/kg in the 0–5 cm depth interval of the sediment core to 100–115 Bq/kg at a depth of 55–58 cm, with positive and negative ^210^Pb deviations from exponential function. These small changes could be induced by slightly variable sediment mass accumulation rate (SMAR) and consequently more pronounced ^210^Pb dilution for intervals with higher SMAR values and ^210^Pb concentrating effect for intervals with lower SMAR values (S1 Fig). These deviations were accounted in calculation of partial sedimentation rates for certain depth (S2 Fig).

Below the depth of 58 cm, the specific activity of ^210^Pb within the limits of uncertainties was close to the maximal level of ^214^Pb in sediments, which corresponds to the supported ^210^Pb level. Based on the conventional statistics (^210^Pb average value in sediment core plus 3 standard deviations), the maximal level of ^214^Pb in sediments was evaluated to be 85 Bq/kg.

The chronology based on ^210^Pb for the second half of the 20th century was validated by using data on the artificial radionuclide ^137^Cs fallouts from the atmosphere due to the Chernobyl NPP accident and nuclear weapons testing in the atmosphere, as independent chronostratigraphic markers.

The depth profile of the ^137^Cs activity in sediments was in good agreement with the ^210^Pb CRS dates (S3 Fig). Two peaks of ^137^Cs at the sediment depths of 49 and 33 cm corresponded to the ^210^Pb dates of ~1963.2 ± 2.2 and ~1986.1 ± 1.0 AD. Below the 49 cm depth, ^137^Cs activity concentration in sediments rapidly decreased. At the bottom of the core (59–60 cm depth slice), it was 7.4 ± 2.8 Bq/kg. Below the depth of 58 cm, which corresponds to the calendar date of 1950 ± 10 AD, the ^210^Pb chronology is uncertain.

Details on ^210^Pb and ^137^Cs activity profiles as well as the age-depth model and sedimentation rate data are given in the supporting information (S1 Text, S1 and S2 Figs).

### Sample pretreatment and measurements

Radiocarbon specific activity measurements were performed in two organic fractions of the lake sediment: alkali-soluble and organic matter that is not soluble in alkali. In agreement with Kleber and Lehmann’s argument that alkaline extraction cannot separate humic and non-humic substances [26], we decided not to use the terms of humus, humic acids, and other subcategories of humic substances.

Alkali-soluble and alkali-insoluble organic fractions in sediment samples were extracted using the acid-base-acid (ABA) method, including sequential washes with 1 M HCl (overnight), 0.2 M NaOH (for 1 h), and 1 M HCl (for 1 h) to yield the alkali-insoluble fraction [36]. The *Coregonus albula* scales (S1 Table), obtained from sets of 5 mature fishes (age of the third year (2+ y)) caught in summer in 1980–1999 and 2005–2012, were decalcified by immersing them into 1.2 N HCl for 2 min [37]. In doing research for this work, we did not work with living organisms. We measured ^14^C specific activity in fish samples already collected (8–30 years ago) and stored at -40 ^o^C until analysis. All samples were graphitized using Automated Graphitization Equipment AGE-3 (IonPlus AG) prior measurements with a 250 kV single stage accelerator mass spectrometer (SSAMS, NEC, USA) at the Center for Physical Sciences and Technology in Vilnius. The background of measurements was estimated to be 2.45 × 10^−3^ f_M_ (fraction of modern carbon) using phthalic acid (Merck, cat. #822298). The IAEA-C3 standard was used as a reference material (the percent of a modern carbon (pMC) value of 129.41). The ^14^C/^12^C ratio was measured with an accuracy better than 0.3%.

The measurements are reported in units of pMC [38,39]:

$${}^{14}a^{s}=\frac{A_{SN}}{A_{ON}}\times 100\%,$$ (1)

where ^14^*a^s^* is ^14^C specific activity, *A*_*SN*_ is the specific activity of the sample *A*_*S*_, normalized to δ^13^C = −25‰; *A*_*ON*_ is the normalized specific activity of the standard.

The freshwater reservoir effect (FRE) is defined as the difference between the radiocarbon isotope ratio (^14^C/^12^C) in the terrestrial and freshwater bodies. The radiocarbon reservoir age (RRA) was calculated according to the following equation [40]:

$$RRA=8033\times \ln(\frac{{}^{14}a_{T}^{S}}{{}^{14}a_{A}^{S}}),$$ (2)

where ${}^{14}a_{T}^{S}$ and ${}^{14}a_{A}^{S}$ is ^14^C specific activity in the terrestrial and aquatic samples of the same period, respectively. As ${}^{14}a_{T}^{S}$, for the period before 1957, we used atmospheric ^14^C specific activity data from OxCal v4.2.4 [41,42]; for the period of 1957–2013, we used ^14^C specific activity measurements in *P*. *sylvestris* from the background location and from the surroundings of INPP [17].

The total nitrogen (TN) (%) and total organic carbon (TOC) (%) content (for TOC/TN or so-called C/N ratio determination) in the sediments were measured using an elemental analyzer (Thermo Flash EA 1112). Standards sulphanilamide (Merck, cat. #111799) and nicotinamide (Sigma Aldrich, cat. #72345) were used for calibration. The long-term standard measurements were performed with a precision of <1.1% for TOC and <0.6% for TN. Before analysis, the sediment samples were acidified with 1 N HCl to remove carbonates, were then rinsed in de-ionized water to a neutral pH, and re-dried.

## Results and discussion

### Radiocarbon specific activity in sediments

**Fig 2:**
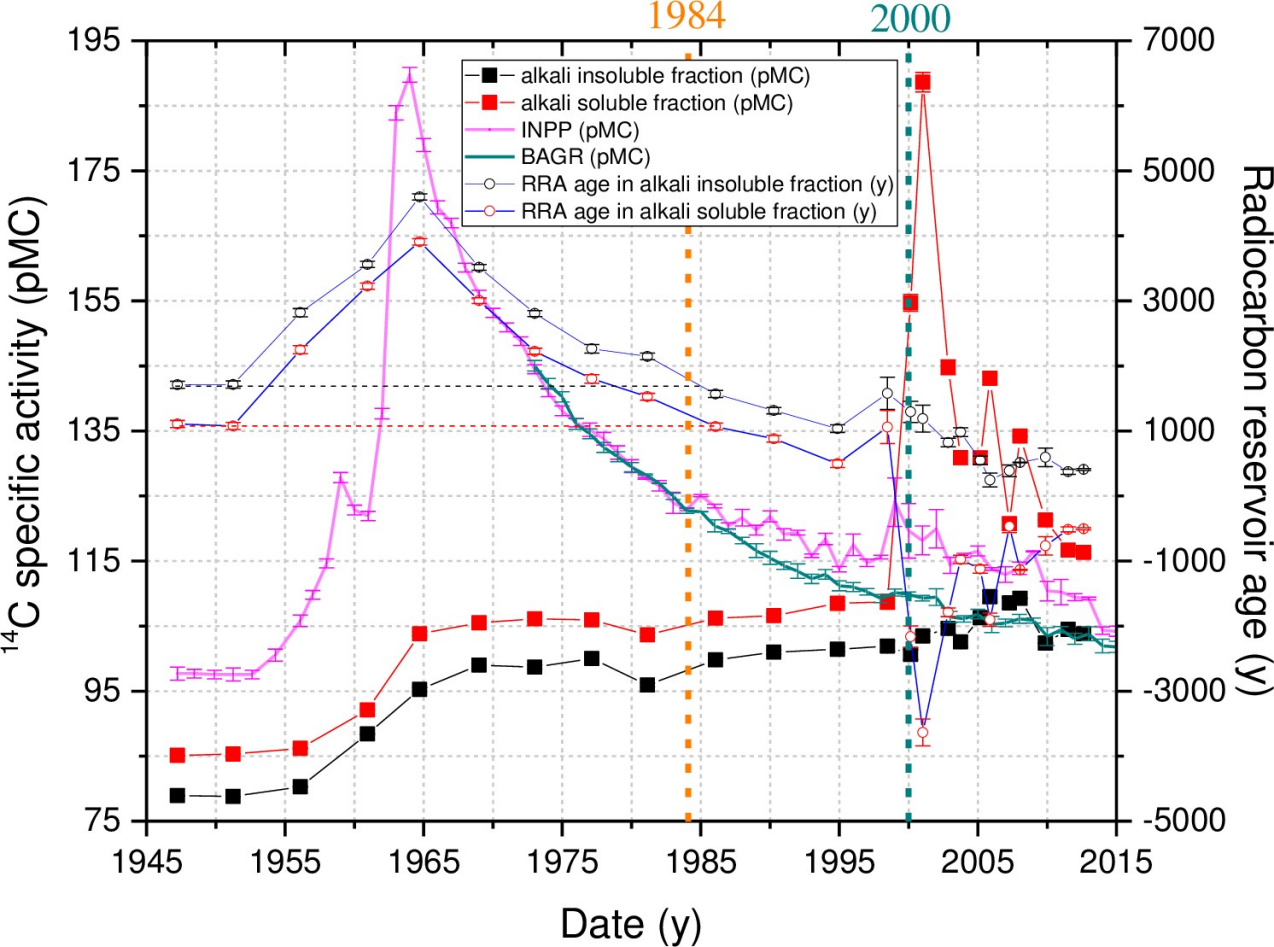
^14^C specific activity in the alkali-soluble and alkali-insoluble sediment fractions, as well as in *P*. *sylvestris* tree rings from the INPP area (INPP) and the background area (BAGR) located 165 km southwest from the INPP (data taken from [27]). The plotted radiocarbon reservoir age (RRA) data was established (using Eq 2) in both alkali-soluble and -insoluble fractions.

Corresponding results for both organic fractions are shown in Fig 2. ^14^C specific activity in the alkali-insoluble organic fraction ranged from 78.8 to 109.5 pMC, whereas ^14^C specific activity in the alkali-soluble organic sediment fraction varied in the 85.1–188.6 pMC interval over the 66-year period (1947–2013) under investigation.

### Pre- operational period (1947–1983)

In 1953, the hydroelectric power plant (HEPP) was built on the Prorva River, the only outflow from Lake Drūkšiai. This resulted in an increase of the lake water level by 0.3 m. In the same year, the entire Apyvardė River flow was directed to Lake Drūkšiai by damming the Drūkša River downstream the Apyvardė River (Fig 1). As a result, the Lake Drūkšiai catchment area increased by 24% [43]. This event was one of the reasons for the increase in the sediment mass accumulation rate (S2 Fig). However, the increased water level did not result in significant changes in water and atmospheric CO_2_ exchange rates, since ^14^C specific activity was unchanged in both sediment fractions (in average 85.54 ± 0.58 pMC and 79.34 ± 0.81 pMC in alkali-soluble and -insoluble fractions, respectively) from 1947 until the beginning of the nuclear weapons tests. Nuclear weapons tests caused an increase in 14C specific activity in both sediment fractions by 19.9 pMC. Whereas in the atmosphere, ^14^C specific activity increased by 95 pMC. This means that atmospheric carbon contributes only about 20% of the total carbon accumulated in the sediments. This also led to an increase in RRA in both fractions by 2850 ± 82 y. The RRA values before 1955 were 1092 ± 84 y and 1913 ± 82 y in alkali-soluble and -insoluble fractions, respectively.

### Early operational period (1984–1999)

The INPP started its operational activity in December 1983. Accordingly, the radiocarbon specific activity in both fractions started to increase.

From 1947 to 1999, the radiocarbon specific activity in both fractions exhibits the parallel course, only ^14^C concentration in the alkali-soluble fraction being 5 ± 1 pMC higher than in the alkali-insoluble fraction.

The C/N ratio values of 10÷12 (S4 Fig) indicate that the main contributors to sediment organic matter are aquatic plants fixing in water dissolved inorganic carbon (DIC). The terrestrial origin organic matter contribution to the sediments did not exceed 16–22% (calculated assuming that the C/N ratio was 6.6 and 40 in phytoplankton and vascular plants, respectively) [44–47].

The DIC concentration and the isotope composition depend on various factors affecting lake water and atmospheric CO_2_ exchange rates, such as organic and inorganic carbon transportation [48,49], the rates of organic carbon production, mineralization, and carbonate weathering and dissolution [50–54].

In 1982, the HEPP ceased electricity production, but the dam was still used for maintaining a high level of lake water required for cooling the INPP; i.e., the regime of watershed hydrology of Lake Drūkšiai basically remained unchanged. The mean water level fluctuation amplitude was kept at ~ 0.2–0.6 m to maintain safety of water use and preservation regulations, stating that the annual lake water level fluctuation amplitude should not exceed 1.2 m in order to reduce the hazards of shore abrasion [43]. This means that changes in the ^14^C specific activity in lake objects were not caused by the lake watershed hydrology affecting organic and inorganic matter transportation and water residence time [8,55,56].

**Fig 3:**
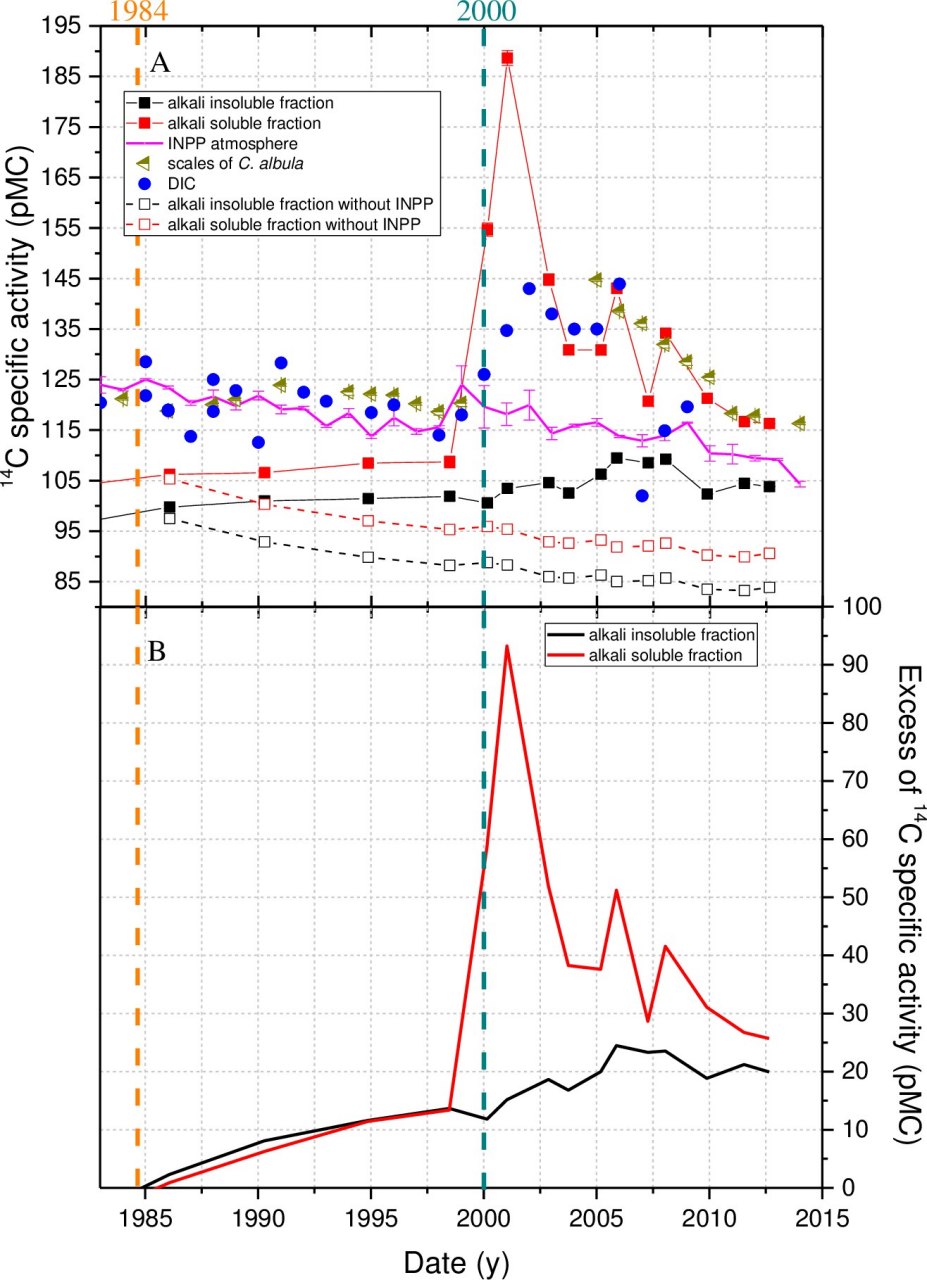
(A) ^14^C specific activity in the alkali-soluble and alkali-insoluble sediment fractions, in the scales of *C*. *Albula* and in DIC (data from [29]) during the period of INPP operation as well as radiocarbon specific activity that would have occurred without any INPP impact (data was obtained by Eq 2, assuming the constant RRA value until the bomb peak; ^14^C specific activity data for terrestrial samples were taken measurements in *P*. *sylvestris* from the background location (BAGR)). (B) Evaluated INPP impact in terms of excess of ^14^C specific activity (in pMC) in the alkali-soluble and -insoluble sedimentary organic fractions.

^14^C specific activity measurements in DIC in Lake Drūkšiai were performed once or twice a year from 1980 to 2009 [29]. Until 2000, the trend of ^14^C specific activity in DIC [29] coincided with the corresponding year radiocarbon measurement in tree rings, sampled from the *P*. *sylvestris* trees in the vicinity of the INPP (see Fig 3) [27].

Measurements showed that, until 2000, the values of ^14^C specific activity in the *C*. *albula* scales within ±3 pMC corresponded to the values of ^14^C specific activity in Lake Drūkšiai DIC (Fig 3A). It was proved in earlier investigations that the *C*. *albula* feeds exclusively on zooplankton [57,58]. Thus, the bioaccumulation of ^14^C in higher trophic levels through a relatively short food chain (DIC → aquatic primary producers → zooplankton → *C*. *albula*) is expected. The existence of such a semi-separate pelagic food chain was revealed by previous stable isotopes studies in Lake Drūkšiai [59]. The age of the caught *C*. *albula* was ~2 y. This mean that the *C*. *albula* scales reflect averaged over ~2 y ^14^C specific activity values in the DIC of the lake water for this period.

Therefore, it can be stated that, until 2000, the main source of ^14^C pollution was in DIC form. As can be seen in Fig 3A, both sediment fractions followed the ^14^C specific activity change in the DIC and in fish scales. No changes in the distribution of radiocarbon in both fractions were also observed during this period.

The RRA in both fractions regained its former pre-bomb values at the beginning of the INPP operation in 1986–1987. From the start of the INPP operation up to 1995, RRA values in sediment fractions decreased by 537 ± 79 y. However, from 1995 to 1999, the RRA in both factions increased by 540 ± 63 y. In 1998–1999, an increase in ^14^C specific activity (up to 10 pMC) was also observed in the tree samples from the vicinity of the INPP [27] (Fig 2). Enhanced maintenance activities are characteristic for the period 1998–2003. During this period, 245 fuel channel zirconium-niobium alloy tubes were extracted from the reactor cores and replaced by the new ones. During the replacement procedure the reactor cavity containing the graphite stack is opened and increased releases of ^14^CO_2_ are expected through the ventilation system of the plant.

Radiocarbon specific activity characteristic to Lake Drūkšiai with the assumption of no INPP impact was calculated for both sediment fractions (Eq 2) supposing that the RRA in both fractions had pre-bomb values (Fig 3B). Thus, during 1984–1999, excess of ^14^C specific activity in both fractions reached 11.4–13.6 pMC. A similar increase in ^14^C specific activity during this period should have been in DIC, as aquatic plants assimilating DIC are the main sedimentary organic source. No measurement data were collected for the period before 1980, so we can only determine the increase in ^14^C specific activity in DIC after the INPP started to operate. Characteristic DIC concentration value in the lake used to be ~2 mM. A rough estimate showed that, during this period, the pollution from the INPP to Lake Drūkšiai used to be up to 0.35–0.41 GBq of ^14^C per year. It includes both airborne and DIC discharges to the lake from the INPP. Assuming a 20% contribution of the airborne pathway for that period would only be a rough approximation due to changes in the CO_2_ exchange rate between the air and lake water caused mainly by the industrial impact of the INPP. During the period of operation for both reactors (i.e. 1987–2004), the water surface temperature in some parts of the lake increased to 32–35 ^o^C during the summer months because of the thermal load [60]. Due to the elevated temperatures of the lake combined with excessive pollution by nitrogen and phosphorus delivered with the sewage water of the town of Visaginas, the trophic state of the lake has changed from (oligo) mesotrophic to almost eutrophic [30].

### Late operational period (2000–2013)

The period from 2000 to 2013 is distinctive by a considerable increase of ^14^C specific activity in alkali-soluble fraction. During the period from 2000 to 2002 (Figs 2 and 3A, S1 Table), a sharp increase of ^14^C specific activity by ~80 pMC was observed only in the alkali-soluble organic fraction, whereas a substantially smaller increase (by ~4 pMC) of radiocarbon content can be seen in the alkali-insoluble fraction. During this period, radiocarbon specific activity values increased in DIC by 25 pMC. It must be taken into account that water samples were previously taken only once or twice a year, mostly at Sampling Station No. 6 (55° 34' 33" N, 26° 37' 15" E, Fig 1). The results of the measurements in DIC show ^14^C specific activity at that particular site only at the time of sampling. These ^14^C values in DIC samples of the period 2000–2002 could differ from sediments sampled at the Station No 1, reflecting averaged 2–3 y DIC values of that particular place. Spatial variation in the lakes can result in ^14^C age differences within the same type of samples of up to several thousand years [8,61]. Unfortunately, we do not have any fish samples from the period of 2000–2004, which could reflect averaged ^14^C specific activity in the DIC averaged over 2–3 y.

During the periods of 2005–2006 and 2008–2009, two ^14^C specific activity peaks of 143.1 and 134.2 pMC, respectively, can be observed in the alkali-soluble fraction. Meanwhile, radiocarbon specific activity increased up to 109.3 pMC (by 7 pMC) only in the alkali-insoluble fraction during both periods. In the DIC fraction, a corresponding increase of up to 143.9 pMC was noted.

In fish scale samples taken in 2005–2012, ^14^C specific activity corresponded well to radiocarbon change patterns in the alkali-soluble fraction for that period, but it was quite different from the values of ^14^C specific activity in DIC. Samples from the 1985–1999 period showed (Fig 3A) ^14^C specific activity differences in fish samples (as well as in DIC) and alkali-soluble fractions up to 12.7 ± 2.1 pMC (and 10.6 ± 3.5 pMC if DIC is included when averaging). This indicates that, during the 2000–2009 period, there were active ^14^C introduction processes other than DIC → aquatic primary producers → sediment fractions. In case the main source of ^14^C pollution was DIC, both sediment fractions would follow the DIC ^14^C specific activity trend.

The most plausible explanation for this phenomenon could be that, during this period, the INPP discharged elevated levels of ^14^C incorporated into some specific water-soluble organic compounds used in the technological processes of NPP operation and maintenance. This additional ^14^C may have been transmitted to the alkali-soluble sediment organic fraction. Earlier investigations showed that low molecular weight (<3.5 kDa) xenobiotics [62,63] and their metabolites [50] can be easily taken up and bioconcentrated by freshwater plants and organisms. Dietary uptake contributed little (up to a few percent) to overall uptake [64]. This may have been the reason for the same ^14^C specific activity trend in the alkaline-soluble sediment organic fraction and fish samples in 2005–2013.

Differences in ^14^Cspecific activity in both sediment fractions indicate that, during the 2000–2002 and 2006–2008 episodes (Figs 2 and 3A), the sources of ^14^C were of a different origin and chemical nature. The unusual trends of ^14^C specific activity in the bottom sediments reveal new paths of ^14^C ingress into the lake ecosystem, which, compared to cases of routine operation, are much more likely to occur from the zones of accumulation of the technological chains of the INPP under decommissioning. It has to be noted that, in the late operational period, new processes of waste management, treatment, and deactivation have been introduced at the INPP.

## Conclusions

Our study of radiocarbon distribution in organic alkali-soluble and alkali-insoluble sediment fractions of Lake Drūkšiai covers a period of 66 years (1947–2010). We analysed the new ^14^C specific activity data obtained from the measurements of sediment core as well as in *C*. *albula* scale samples. We investigated how the radiocarbon distribution in this fish and DIC coincide due to expected DIC → aquatic primary producers → zooplankton → *C*. *albula* ^14^C accumulation chain. The data of radiocarbon specific activity in the tree rings of the INPP vicinity are also included in our analysis. This allowed establishing a full picture of ^14^C specific activity changes over several decades in the main indicators of INPP environment.

The slight increase in water level of Lake Drūkšiai caused by the hydroelectric power plant constructed in 1953 did not appear to have a significant effect on ^14^C distribution in sediments. C/N measurements show that the main contribution (~78–84%) of organic fraction in sediments throughout the study period is of autochthonous origin. Thus, both before the commissioning of the INPP and during the first 15 years of its operation (until 2000) with minor maintenance activities, the radiocarbon specific activity in both fractions of bottom sediments exhibits the parallel course, and undisturbed chain DIC → aquatic primary producers → sediments governed migration of radiocarbon in Lake Drūkšiai sediments. During this period the ^14^C specific activity values in *C*. *albula* scales and DIC samples were in a good agreement.^14^C redistribution observed in the last 11 years of operation of the INPP in both organic sediment fractions indicates that another ^14^C accumulation pathway has taken place. This might be explained by the new processes of waste management and treatment implemented at the INPP in late operational period before the final shutdown of Unit 1 (end of 2004) and Unit 2 (end of 2009).

The gaseous discharges from the INPP with RBMK type reactors are mainly carbon dioxide containing ^14^C. Although systematic monitoring of ^14^C airborne releases started in 2008 only, it can be relatively well reproduced by ^14^C measurements in tree rings near the INPP. Routine monitoring of radiocarbon in liquid releases was not performed, but it is expected that radiocarbon releases from RBMK type reactors is in dissolved inorganic carbon form. However, complex investigations of the radiocarbon specific activity in alkali-soluble and -insoluble fractions of sediment core layers indicate changes in ^14^C redistributions between sediment fractions as well as discrepancies in radiocarbon specific activity values in the *C*. *albula* scales and DIC. This can retrospectively reveal facts of occurrence of elevated liquid radiocarbon releases, not only in DIC but also in dissolved organic compounds which might have been used in processes of maintenance and decontamination of technological circuits of the plant.

## Supporting information

S1 Fig Lake Drūkšiai sediment records: A) ^137^Cs and ^210^ Pb profiles; B) model ages from ^210^Pb profile.
(PDF)

S2 Fig Sediment Mass Accumulation Rate (SMAR) based on ^210^Pb chronology. (PDF)

S3 Fig ^137^Cs specific activity in sediment 1 cm thick slices. Sediment dating was performed using ^210^Pb CRS model.
(PDF)

S4 Fig C/N ratio values in sedimentary organic matter. (PDF)

S1 Table ^210^Pb, ^214^Pb and ^137^Cs activity data; age of the sediment layers, ^14^C specific activity in sediment Alkali-Soluble (AS) and Alkali-Insoluble (AIS) sediment organic fractions as well as in the *C*. *albula* scales; total length of the *C*. *albula* (TL). Data is shown as mean ± standard deviation.
(PDF)

S1 Text ^210^Pb and ^137^Cs sediment dating and sedimentation rate determination. (PDF)
