# Supplementary figures and images for: Distribution of radiocarbon in sediments of the cooling pond of RBMK type Ignalina Nuclear Power Plant in Lithuania
Source: PLoS One. 2020 Aug 17;15(8):e0237605. doi: 10.1371/journal.pone.0237605 (PMC7430730; doi:10.1371/journal.pone.0237605)

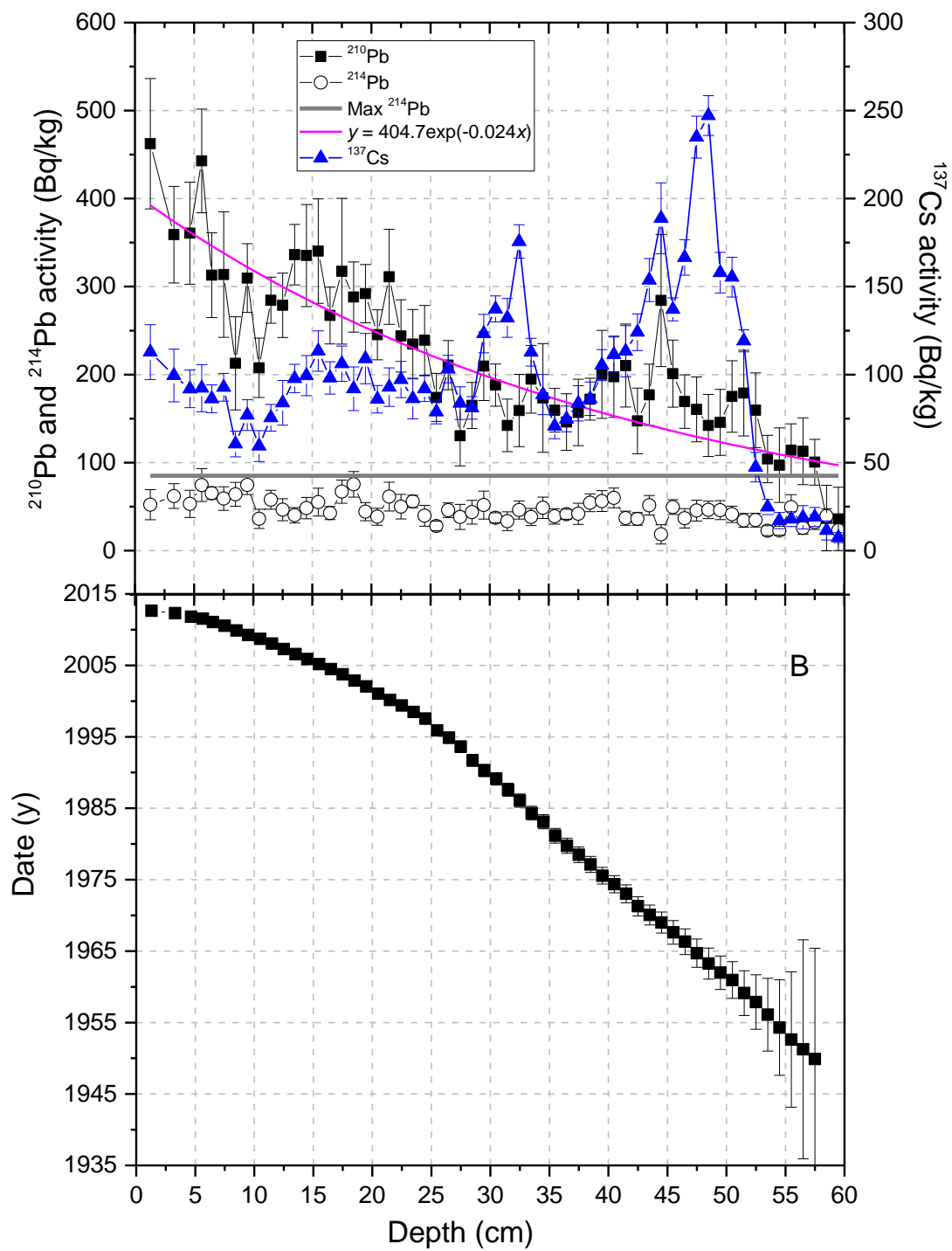

Supplement: S1 Fig — Lake Drūkšiai sediment records: A) 137Cs and 210 Pb profiles; B) model ages from 210Pb profile. (PDF) [file pone.0237605.s001.pdf]

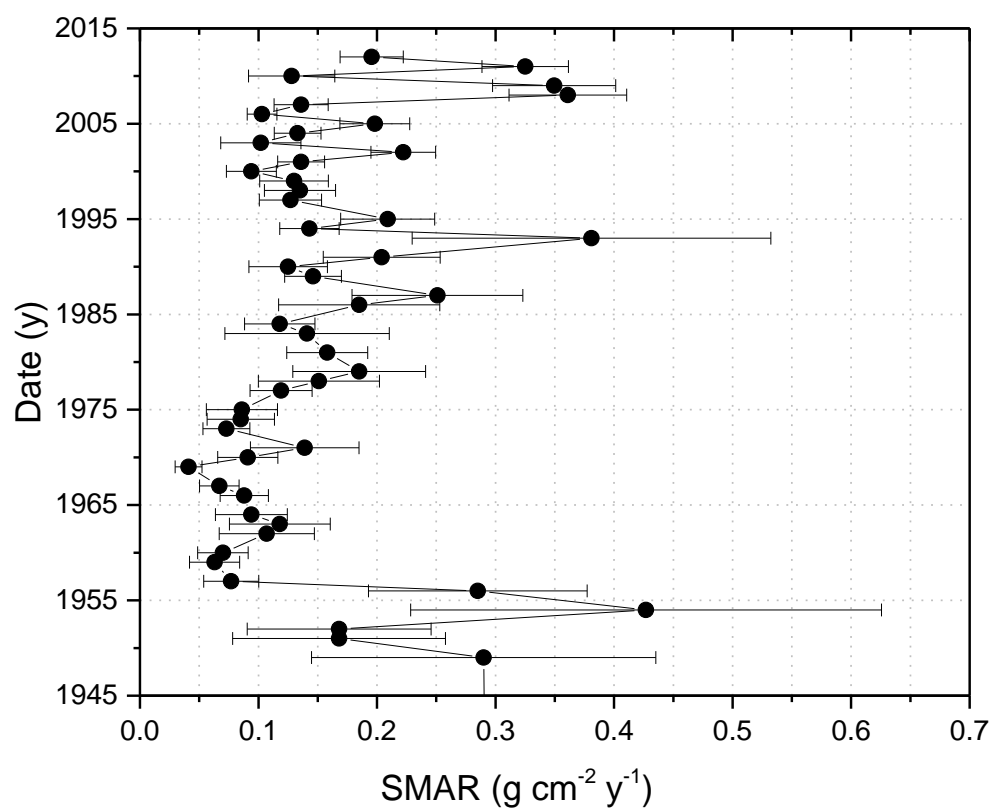

Supplement: S2 Fig — (PDF) [file pone.0237605.s002.pdf]

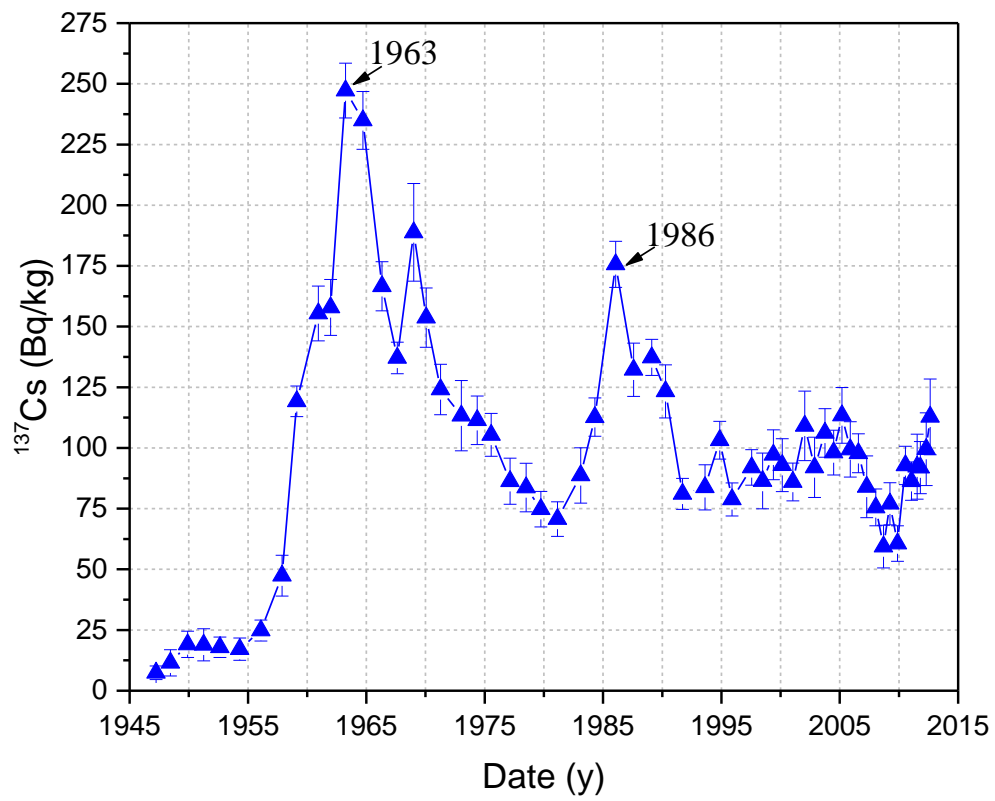

Supplement: S3 Fig — Sediment dating was performed using 210Pb CRS model. (PDF) [file pone.0237605.s003.pdf]

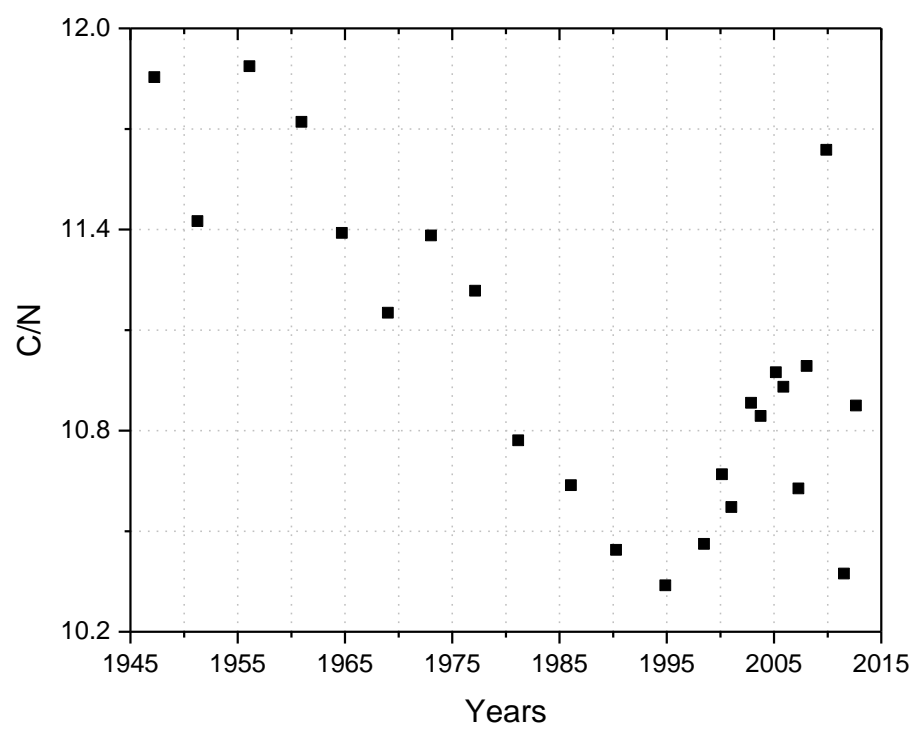

Supplement: S4 Fig — (PDF) [file pone.0237605.s004.pdf]
