# Supplementary material for: Distribution of radiocarbon in sediments of the cooling pond of RBMK type Ignalina Nuclear Power Plant in Lithuania
Source: PLoS One. 2020 Aug 17;15(8):e0237605. doi: 10.1371/journal.pone.0237605 (PMC7430730; doi:10.1371/journal.pone.0237605)
Supplement: S1 Table — Data is shown as mean ± standard deviation. (PDF) [file pone.0237605.s005.pdf]

Table S1. ^{210}Pb, ^{214}Pb and ^{137}Cs activity data; age of the sediment layers, ^{14}C specific activity in sediment alkali-soluble (AS) and alkali-insoluble (AIS) sediment organic fractions as well as in the *C. albula* scales; total length of the *C. albula* (TL). Data is shown as mean ± standard deviation.

Depth (cm)	^{210}Pb activity (Bq/kg)	^{214}Pb activity (Bq/kg)	^{137}Cs activity (Bq/kg)	Age of the sediment layers (y)	^{14}C specific activity in AIS (pMC)	^{14}C specific activity in AS (pMC)	Year the fish was caught	TL (cm)	^{14}C specific activity in fish scales (pMC)
								(n=5)	
1	462.4±74.1	52.1±17.1	112.8±15.6	2012.6±0.3	103.82±0.14	116.31±0.15	2012	20.0 ± 0.7	117.82 ± 0.23
3	359±54.9	62.0±14.1	99.5±15.0	2012.3±0.3			2011	19.7 ± 0.9	118.29 ± 0.23
5	360.7±58	53.1±15.4	91.9±10.7	2011.8±0.4			2010	18.6 ± 0.7	125.46 ± 0.69
6	442.8±58.9	74.5±18.6	92.3±13.4	2011.5±0.4	104.47±0.14	116.67±0.15	2009	18.6 ± 1.0	128.52 ± 0.71
7	313±48.2	65.2±7.2	86.3±7.9	2011.1±0.4			2008	17.3 ± 0.3	131.98 ± 0.73
8	313.6±71.3	59.2±13.4	92.8±7.9	2010.5±0.4			2007	18.4 ± 1.2	136.11 ± 0.75
9	212.8±52.9	64±13.6	60.6±7.3	2009.9±0.5	102.37±0.14	121.30±0.15	2006	19.3 ± 0.8	138.53 ± 0.27
10	309.7±39	74.6±10.8	77.0±8.7	2009.2±0.5			2005	18.9 ± 1.0	144.74 ± 0.28
11	207.7±33.7	36.1±11.0	59.4±8.8	2008.7±0.5			1999	18.8 ± 0.7	120.32 ± 0.66
12	284.5±26.2	57.8±10.7	75.5±7.6	2008.0±0.5	109.27±0.15	134.19±0.06	1998	17.7 ± 0.6	118.63 ± 0.65
13	278.8±36.6	46.5±12.5	84.0±12.7	2007.3±0.5	108.55±0.83	120.72±1.00	1997	17.6 ± 0.6	120.21 ± 0.24
14	336.3±34.4	40.2±8.7	97.8±8.0	2006.6±0.5			1996	18.5 ± 0.8	121.95 ± 0.24
15	335.3±58	48.3±12.1	99.4±11.4	2005.9±0.6	109.50±0.88	143.08±1.00	1995	17.9 ± 0.6	122.16 ± 0.24
16	340.3±59.4	55.1±15.8	113.4±11.5	2005.2±0.6	106.29±0.81	130.85±1.00	1994	17.6 ± 0.5	122.55 ± 0.68
17	267.1±32.3	42.7±7.5	98.1±9.2	2004.5±0.6			1991	18.1 ± 0.8	123.90 ± 0.24
18	317.4±82.8	67.0±13.2	106.2±10.0	2003.8±0.6	102.54±0.82	130.87±1.00	1989	18.4 ± 0.9	121.05 ± 0.24
19	288.1±39.9	75.4±14.5	92.0±12.4	2002.9±0.6	104.61±0.80	144.78±1.13	1988	18.8 ± 0.7	120.05 ± 0.24
20	292±33.1	44.3±10.3	109.1±14.3	2002.0±0.7			1986	19.1 ± 0.9	118.80 ± 0.65
21	245.3±28.4	38.5±9.7	86±7.8	2001.0±0.7	103.47±0.77	188.62±1.48	1984	19.6 ± 1.2	121.20 ± 0.24
22	311.1±54.1	61.4±16.4	92.9±10.9	2000.1±0.7	100.61±0.78	154.69±1.29	1980	19.8 ± 0.5	125.55 ± 0.25
23	243.9±41.1	50.0±14.0	97.2±10.3	1999.4±0.7					
24	234.6±39.4	55.8±7.0	86.4±11.5	1998.5±0.7	101.91±0.78	108.73±1.15			
25	239±39.6	39.6±11.8	92±7.3	1997.5±0.7					
26	174.1±27.3	28.0±5.4	78.8±6.8	1995.9±0.8					

27	210.8±27.9	45.9±8.3	103.2±7.8	1994.9±0.8	101.43±0.32	108.48±0.34			
28	130.5±34.2	38.3±14.2	83.8±9.3	1993.6±0.8					
29	165±26.2	43.7±13.5	81.1±6.4	1991.7±0.8					
30	209.6±39.1	51.8±15.9	123.3±10.9	1990.3±0.9	100.98±0.32	106.58±0.34			
31	188.1±24	37.3±5.7	137.3±7.4	1989.1±0.9					
32	142.3±29.8	33.4±10.7	132.2±11	1987.6±0.9					
33	159.2±41.1	46.1±10.3	175.6±9.5	1986.1±0.9	99.79±0.31	106.22±0.34			
34	194.7±38.5	38.2±9.7	112.7±7.9	1984.3±1.0					
35	173.5±61.7	48.7±12.1	88.7±11.4	1983.1±1.0					
36	159.5±24.8	38.9±8.7	70.7±7.1	1981.2±1.0	95.93±0.30	103.62±0.33			
37	146.1±32.1	41.4±5.7	74.8±7.3	1979.7±1.1					
38	157±37.8	41.8±12.1	83.7±10.0	1978.5±1.1					
39	172±23.8	54.4±10.1	86.3±9.5	1977.1±1.1	100.01±0.32	105.94±0.33			
40	200.3±50.0	56.3±11.8	105.4±8.8	1975.6±1.2					
41	197.5±46.1	59.9±11.2	111.4±10.0	1974.4±1.2					
42	210.3±47.2	36.9±8.0	113.3±14.5	1973.0±1.3	98.69±0.31	106.10±0.33			
43	147.4±37.3	36.0±7.1	124.1±10.4	1971.3±1.3					
44	177±34.8	51.5±11.1	153.7±12.2	1970.1±1.4					
45	284.2±75.1	18.5±10.9	188.8±20.1	1969.0±1.5	99.00±0.31	105.51±0.33			
46	201.1±38.2	49.5±8.3	137.1±6.5	1967.6±1.7					
47	169.4±30.4	36.7±11.0	166.6±10.1	1966.3±1.8					
48	160.5±36.9	45.5±11.9	234.9±11.9	1964.7±2.0	95.26±0.30	103.81±0.33			
49	142.3±35.3	46.3±10.0	247.2±11.3	1963.2±2.2					
50	145.7±37.5	46.0±10.7	157.9±11.5	1962.0±2.3					
51	175.1±40.6	41.3±9.3	155.4±11.3	1960.9±2.6	88.39±0.14	92.11±0.14			
52	178.9±48.7	34.4±7.9	119.2±6.3	1959.1±3.1					
53	159.5±42.4	34.7±7.8	47.4±8.4	1957.9±3.8					
54	104.1±27	22.6±5.6	24.8±4.3	1956.1±5.1	80.28±0.13	86.20±0.14			
55	97.1±42.3	22.7±5.0	17.1±4.6	1954.3±6.7					
56	114.1±30	49.5±14.0	17.9±4.2	1952.6±9.5					
57	112.9±38	25.7±7.0	18.9±6.6	1951.3±15.3	78.80±0.13	85.31±0.14			
58	100.5±26	32.4±8.4	19.1±5.4	1949.9±15.5					

59	37.0±37.2	39.3±8.2	11.5±5.4						
60	36.0±36.0	23.2±8.5	7.4±2.8		78.94±0.13	85.11±0.14			
