# Supplementary material for: Distribution of radiocarbon in sediments of the cooling pond of RBMK type Ignalina Nuclear Power Plant in Lithuania
Source: PLoS One. 2020 Aug 17;15(8):e0237605. doi: 10.1371/journal.pone.0237605 (PMC7430730; doi:10.1371/journal.pone.0237605)
Supplement: S1 Text — (PDF) [file pone.0237605.s006.pdf]

S1 Text. ^{210}Pb and ^{137}Cs sediment dating and sedimentation rate determination

The ^{210}Pb excess, $^{210}Pb_{ex}$, which was used for the sediment age calculation based on radioactive decay, was determined by subtracting the supported ^{210}Pb from the total ^{210}Pb (S1 Fig). The supported ^{210}Pb was assumed to be in equilibrium with ^{214}Pb and ^{226}Ra. The $^{210}Pb_{ex}$ concentration profile in the sediment core up to the depth of 58 cm exhibited a tendency of exponential decrease with the mass depth, d_m ($^{210}Pb_{ex}=247\times\exp(-0.243\times d_m)$; $R^2=0.66$). The mass depth is a product of the wet thickness of the sediment slice and the dry bulk density. From the exponential decrease of $^{210}Pb_{ex}$ and assuming the constant $^{210}Pb_{ex}$ flux the steady-state sedimentation with a mean SMAR value of 0.13 g/cm^2/y was derived. This mean SMAR value was used to calculate the sediment age and partial SMAR values for the studied core up to the depth of 58 cm. The sediment age-depth function derived from the ^{210}Pb CRS model was expressed by the polynomial equation ($y = 0.0106\times x^2 + 0.5516\times x - 2.5902$; $R^2 = 0.9974$).

The partial SMAR values for the past 65 years have been in the range of 0.041 ± 0.011 to 0.43 ± 0.13 g/cm^2/y with a mean SMAR value of 0.13 g/cm^2/y for the entire dated interval of the core (S2 Fig). The mean SMAR value corresponded to the linear sedimentation of wet matter equal to 0.9 cm/y. The first-order errors (2σ) for the SMAR values increased with depth and were in the range of 15-35%, with the largest values found at the sediment depth of 55–58 cm, where ^{210}Pb activities were approaching the minimum detectable activity (MDA) level.
